# Discovery of Phosphatidic Acid, Phosphatidylcholine, and Phosphatidylserine as Biomarkers for Early Diagnosis of Endometriosis

**DOI:** 10.3389/fphys.2018.00014

**Published:** 2018-01-23

**Authors:** Jingjie Li, Yue Gao, Lihuan Guan, Huizhen Zhang, Jiahong Sun, Xiao Gong, Dongshun Li, Pan Chen, Zheng Ma, Xiaoyan Liang, Min Huang, Huichang Bi

**Affiliations:** ^1^Center of Reproductive Medicine, The Sixth Affiliated Hospital, Sun Yat-sen University, Guangzhou, China; ^2^School of Pharmaceutical Sciences, Sun Yat-sen University, Guangzhou, China; ^3^School of Public Health, Guangdong Pharmaceutical University, Guangzhou, China; ^4^Department of Pharmacy, The First Affiliated Hospital, Sun Yat-sen University, Guangzhou, China; ^5^Institute of Population Research, Peking University, Beijing, China

**Keywords:** endometriosis, lipid profiling, UHPLC-ESI-HRMS, eutopic endometrium, early diagnosis

## Abstract

The sensitivity and specificity of clinical diagnostic indicators and non-invasive diagnostic methods for endometriosis at early stage is not optimal. Previous studies demonstrated that abnormal lipid metabolism was involved in the pathological development of endometriosis. Our cross-sectional study included 21 patients with laparoscopically confirmed endometriosis at stage I–II and 20 infertile women who underwent diagnostic laparoscopy combined with hysteroscopy from January 2014 to January 2015. Eutopic endometrium was collected by pipelle endometrial biopsy. Lipid metabolites were quantified by ultra-high performance liquid chromatography coupled with electrospray ionization high-resolution mass spectrometry (UHPLC-ESI-HRMS). Lipid profiles of endometriosis patients at early stage (I–II) was characterized by a decreased concentration of phosphatidylcholine (18:1/22:6), (20:1/14:1), (20:3/20:4), and phosphatidylserine (20:3/23:1) and an increased concentration of phosphatidic acid (25:5/22:6) compared with control. The synthesized predicting strategy with 5 biomarkers has a specificity of 75.0% and a sensitivity of 90.5%. Lipid profile of eutopic endometrium in endometriosis was effectively characterized by UHPLC-ESI-HRMS-based metabolomics. Our study demonstrated the alteration of phosphatidic acid, phosphatidylcholine, phosphatidylserine metabolites in endometriosis and provided potential biomarkers for semi-invasive diagnose of endometriosis at early stage.

## Introduction

The prevalence of endometriosis is estimated as 2–10% in the general female population and up to 40% in women with subfertility (Eskenazi and Warner, 1997; Ozkan et al., 2008; Dunselman et al., 2014), which significantly compromises quality of life in women and adolescents and causes a substantial societal economic burden (Gao et al., 2006; Nnoaham et al., 2011; Soliman et al., 2016). Currently, diagnosis of endometriosis is extremely challengeable due to similar symptoms to other gynecological and gastrointestinal diseases. Since endometriosis at early stage lacks specific imaging features, the reliable way to diagnose endometriosis, especially at early stage, is surgical laparoscopy. As the common diagnostic indicator, cancer antigen 125 (CA125) has been shown to be more beneficial for diagnosing advanced stages (III–IV) than stages I and II (Hirsch et al., 2016). Therefore, diagnosis of endometriosis is typically delayed up to 8–10 years from the initial appearance of symptoms (Greene et al., 2009; Nnoaham et al., 2011; Hudelist et al., 2012). It is urgent to development novel diagnostic biomarkers and non-invasive methods for endometriosis diagnosis. In this study, we investigated alterations of lipid profile in eutopic endometrium of endometriosis patients at stage I–II by ultra-high performance liquid chromatography coupled with electrospray ionization high-resolution mass spectrometry (UHPLC-ESI-HRMS)-based metabolomics, which provided potential markers for early diagnosis of this disease.

## Materials and methods

### Study design and sample source

A cross-sectional study was performed on stored samples prospectively collected from women who participated in a previous metabonomic study. Participants were recruited from Reproductive Medicine Research Center, Sixth Hospital of Sun Yat-sen University from June 2014 to January 2015, who underwent diagnostic laparoscopy and hysteroscopy for infertility. This study was approved by institutional review board from sixth Hospital of Sun Yat-sen University, and written informed consent was taken from all participants (approval number: G2012021). All participants had regular menstrual cycles (28 ± 7 days) without hormonal treatment in 3 months prior to sample collection. Women laparoscopic and hysteroscopic diagnosed with endometriosis at stage I–II were further visually confirmed the presence of endometriosis according to American Society of Reproductive Medicine revised system (American Society for Reproductive Medicine, 1997). Patients who were diagnosed as endometriosis by transvaginal ultrasonography or had the history of abnormally increased CA125 previously were excluded from this study. Endometrial polyp, endometritis, submucous myoma, and hydrosalpinx were also excluded. Eutopic endometrium samples were collected by pipelle endometrial biopsy on day 3–5 after the end of menstrual bleeding during laparoscopy and hysteroscopy or within 3 months after surgery.

### Sample preparation for lipidomics

Endometrial tissues were obtained from 20 infertile women (Control) and 21 women with endometriosis. Lipids extraction was performed according to methyl-tert-butyl ether (MTBE) method (Matyash et al., 2008). 0.01 g endometrium samples were thawed and then homogenized in 200 μL PBS using Precellys 24 homogenizer (Bertin, France). Then 150 μL of homogenate was added to 1.2 mL chilled mixture of methanol/MTBE/water (4:5:5, v/v/v). Samples were incubated on ice for 1 h and vortexed for 1 min every 15 min. Following centrifugation (2,000 rpm, 5 min), 200 μL supernatant of each sample was transferred to new tubes and dried under nitrogen flow at room temperature. Samples were re-suspended in 500 μL mixture of methanol/isopropanol (1:1, v/v) and centrifuged at 18,000 × g for 5 min at 4°C Finally, 2 μL of supernatant was injected for UHPLC-ESI-HRMS (Thermo Scientific, San Jose, CA) analysis. Three microliter of each sample was mixed to be quality control (QC) samples (Zhang et al., 2017).

### UHPLC-ESI-HRMS measurement of endometrial tissues

Samples were separated using an Ascentis Express C18 2.7 μm column (100 × 2.1 mm, Sigma-Aldrich, St. Louis, MO) on a Thermo Scientific Dionex Ultimate 3000 UHPLC system. Flow rate was 0.3 mL/min while column temperature was 45°C. The mobile phases consisted of (A) 5% acetonitrile in isopropanol with 10 mM ammonium formate and 0.1% formic acid and (B) 50% water in acetonitrile with 10 mM ammonium formate and 0.1% formic acid. Linear gradient was as follows: 0–0.5 min remaining at 20% A, linearly increasing to 50% A at 7.5 min, then linearly decreasing to 20% A at 10 min, following increasing to 100% A at 20 min, holding at 100% A until 21.9 min, then linearly increasing to 80% B at 22 min and equilibrating until 25 min. Mass spectrometry was performed with a Thermo Scientific Q ExactiveTM benchtop Orbitrap mass spectrometer equipped with heated ESI source in ESI positive and negative modes (Thermo Scientific, San Jose, CA). The main parameters for MS/MS included AGC target 1e5; maximum IT 65 ms; isolation window 1.2 *m/z*; normalized collision energy 25, 35 eV in positive mode and 20, 30, and 40 eV in negative mode; apex trigger 5–10 s; and dynamic exclusion 10.0 s. Ionization conditions were operated at spray voltage 3.5 kV and capillary temperature 300°C (Yu et al., 2016).

### Lipidomic data processing

The acquired total ion chromatograms (TIC) and mass spectra from UHPLC-ESI-HRMS were exported as raw files by Xcalibur (Thermo Scientific, San Jose, CA). Three-dimensional data set including *m/z*-values, retention times, and peak areas were extracted by LipidSearch software (Thermo Scientific, San Jose, CA) to perform lipids identification and evaluate matching degrees by A, B, C, D four grades. Orthogonal projection to latent structures discriminant analysis (OPLS-DA) in positive and negative modes were carried out by SIMCA-P 13.0 Software (Umetrics, Kinnelon, NJ) to visualize differences of lipid metabolites between endometriosis patients and control. S-plots were generated in OPLS-DA mode and potential markers were selected on the basis of a variable importance in the projection (VIP) with threshold of 1.0 (Dutta et al., 2016). A logistic regression analysis was used to assessing the strength of association between lipid metabolites and minimal-mild endometriosis. The receiver operating characteristic (ROC) curve was plotted and the area under the curve (AUC) was calculated. The optimal point on ROC curve provided the best trade-off between sensitivity and specificity. Shapiro–Wilk test was used to evaluate the normality of distribution, then statistical significance was calculated using Student's *t*-test and non-parametric Mann–Whitney U-test, with *p* < 0.05 as statistical significance level. Statistical test was carried out by SPSS 20.0 software (IBM Analytics, USA).

## Results

The endometriosis group was composed of 14 patients at stage I and 7 patients at stage II. None of patients in this group was confirmed with ovarian endometriomas by laparoscopy. The control group consisted of 20 participants. Two groups were balanced in terms of age, BMI, and AMH level (Table 1).

**Table 1:** Characteristics of participant.

	**Endometriosis patients (*n* = 21)**	**Control group (*n* = 20)**	***P***
Age (years)	29.71 ± 3.117	30.45 ± 3.034	0.4487
BMI (kg/m^2^)	20.76 ± 1.716	21.24 ± 2.882	0.5198
AMH (ng/ml)	4.417 ± 2.990	6.117 ± 5.079	0.2189
Endometriosis stage			
I stage	14	N/A	
II stage	7	N/A	
Ovarian endometriomas	N/A	N/A	

**Figure 1:**
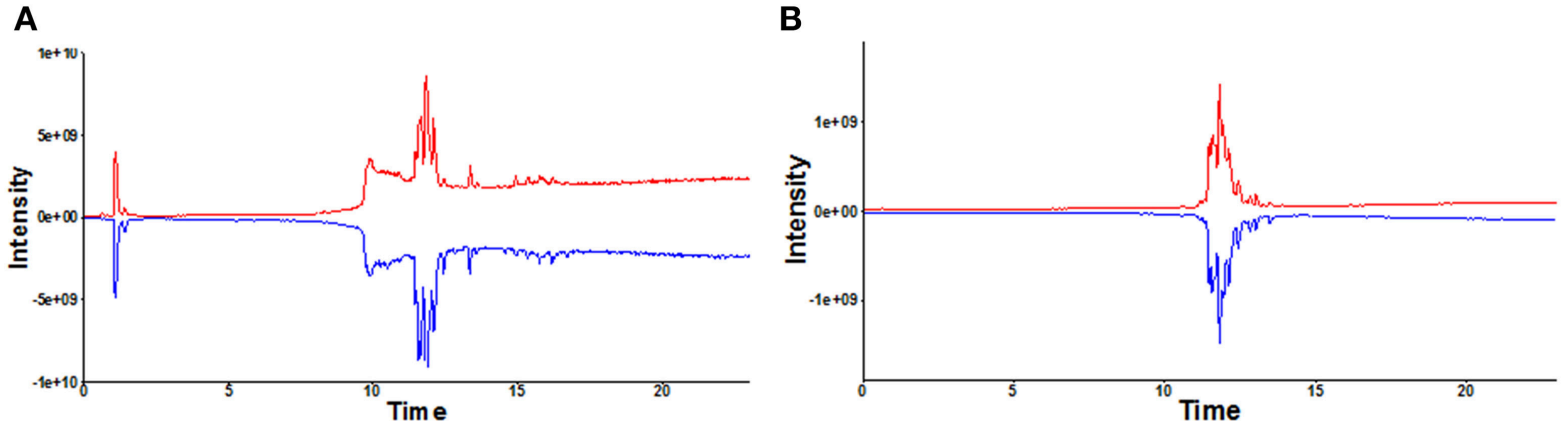
Mirror plot of representative total ion chromatograms (TIC) under positive **(A)** and negative mode **(B)**. TIC in red: healthy controls; TIC in blue: endometriosis patients.

**Figure 2:**
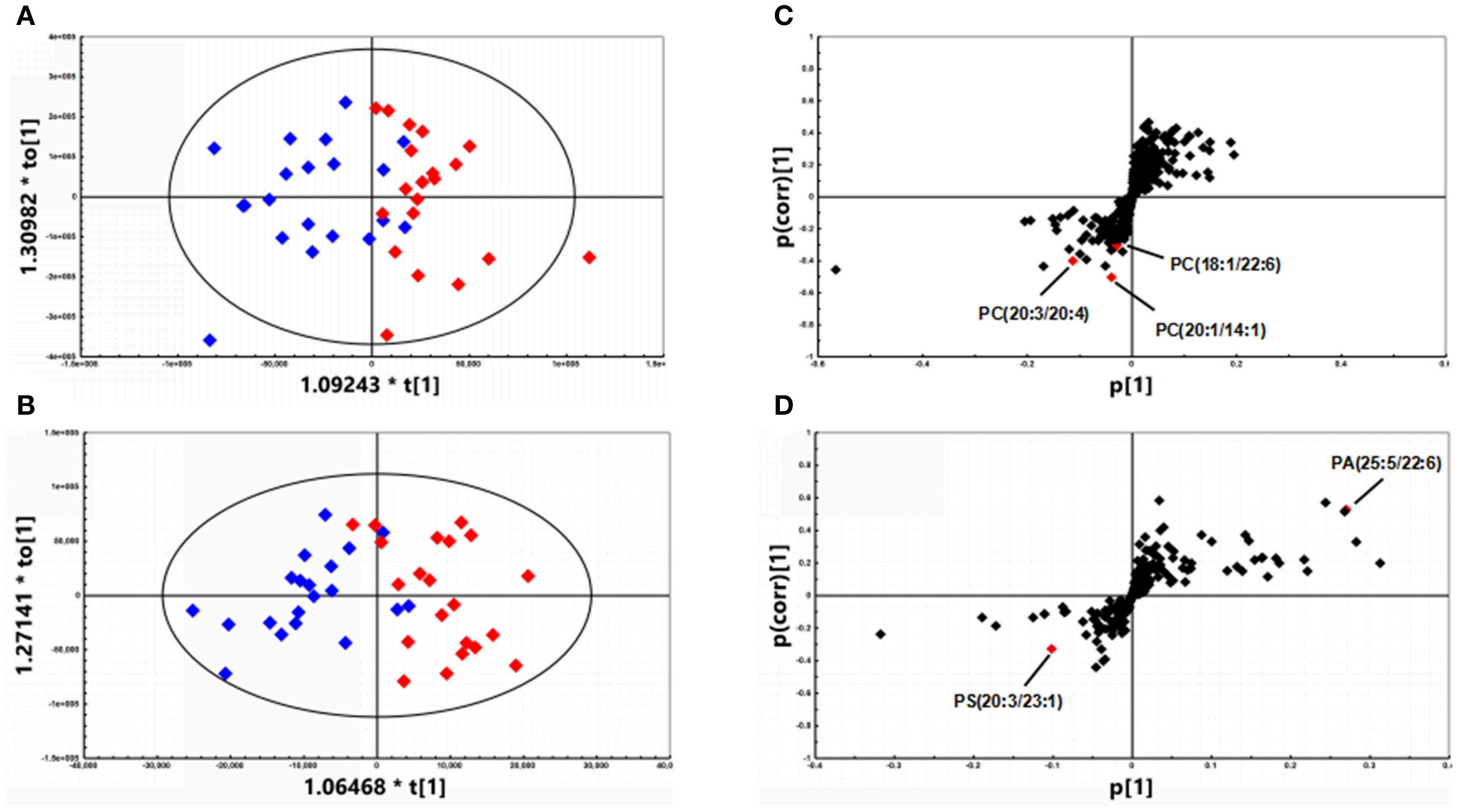
OPLS-DA plots of patients with endometriosis (*n* = 21, *red diamonds*) and healthy controls (*n* = 20, *blue diamonds*) under **(A)** positive (R^2^Y = 0.712; Q^2^ = 0.412) mode and **(B)** negative (R^2^Y = 0.728; Q^2^ = 0.602) mode. **(C)** S-plot under positive mode with selected PCs highlighted in red. **(D)** S-plot under negative mode with selected PE and PA highlighted in red.

**Figure 3:**
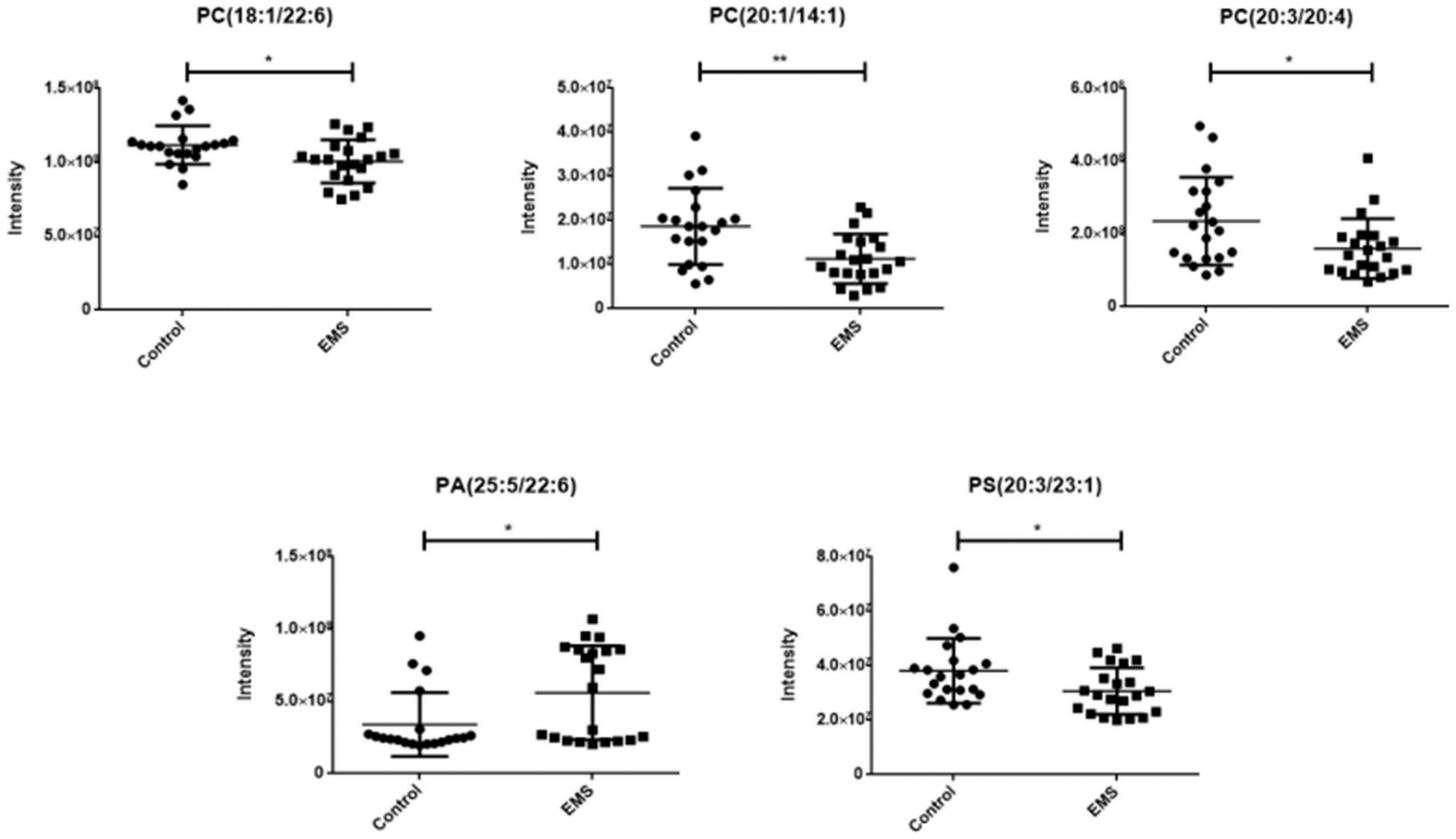
Scatter diagram of five selected lipids. PCs and PS in endometriosis patients were significant decreased compared to healthy controls, while PA in endometriosis patients was higher than healthy controls. Data are expressed as mean ± SD. ^*^p < 0.05, ^**^p < 0.01, endometriosis patients (EMS, *n* = 21) vs. healthy controls (Control, *n* = 20).

**Figure 4:**
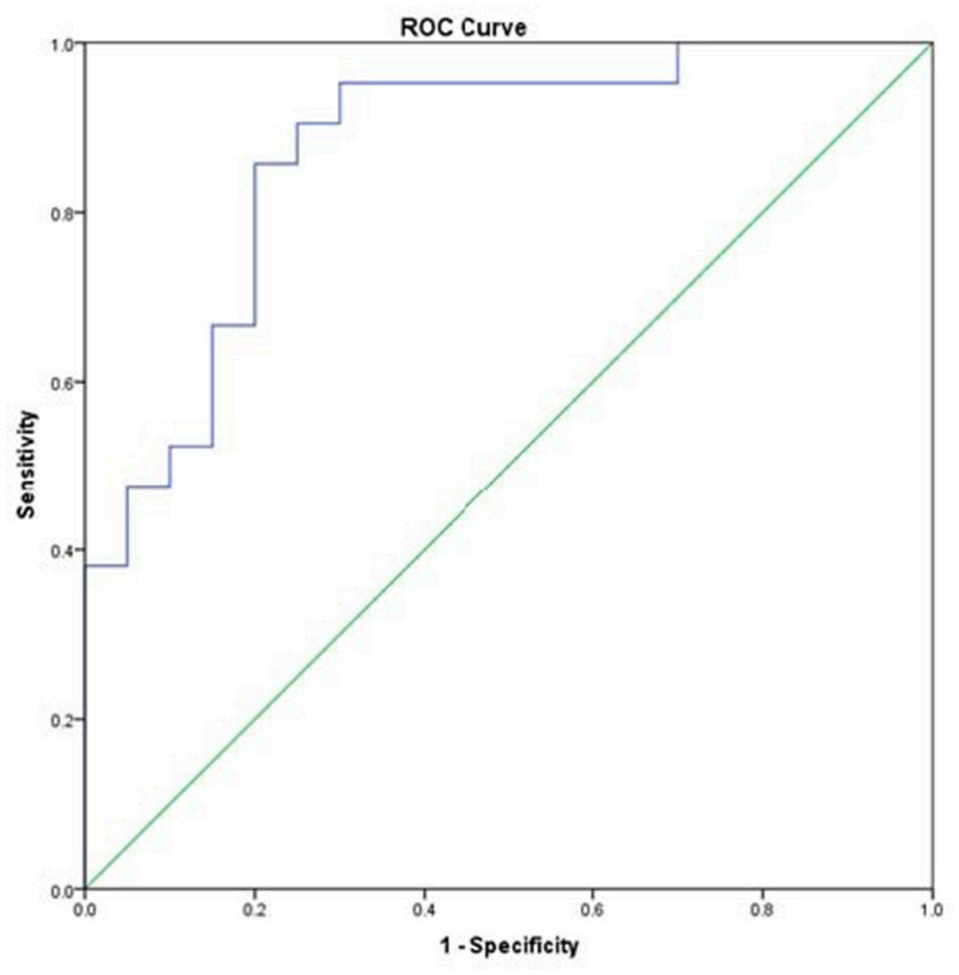
Receiver operating characteristic curves for endometriosis at minimal-mild stage.

A total of 468 ions in positive mode and 253 ions in negative mode were observed. The comparison of TIC can be seen in the mirror plots (Figure 1). Multiple differences in peak intensities detected at the same retention time, indicates different lipid spectrums between endometriosis patients and control. OPLS-DA models with acceptable R^2^Y and Q^2^ revealed a trend of separation between two groups (Figures 2A,B), indicating distinct lipid profiles of endometriosis patients. The alteration of lipids was identified by VIP >1.0 and then validated at an univariate level by Student's *t*-test or Mann–Whitney U-test, with *p* < 0.05 set as the level of statistical significance. Lipid identification was performed using LipidSearch software to directly identify lipid species from accurate precursor *m/z* and MS/MS raw data with the reference of large-scale database. As shown in Figures 2C,D, PC (18:1/22:6), PC (20:1/14:1), PC (20:3/20:4), PA (25:5/22:6), and PS (20:3/23:1) were selected as potential markers and details were summarized in Table 2. Apparently, levels of PC (18:1/22:6), PC (20:1/14:1), PC (20:3/20:4), and PS (20:3/23:1) were significantly decreased in endometriosis group, which have previously been to involve in the progress of endometriosis (Vouk et al., 2016). However, PA (25:5/22:6) level was much higher in endometriosis group compared to control (Figure 3), which could be a novel marker of endometriosis and further facilitated to the mechanism studies of the disease. The multivariable regression model for early stage endometriosis included five independent predictors: PC (18:1/22:6), PC (20:1/14:1), PC (20:3/20:4), PS (20:3/23:1), and PA (25:5/22:6). The apparent AUC of ROC curve for the complete model predicting early endometriosis was 0.871 with a sensitivity of 0.905 and specificity of 0.750 (Figure 4).

**Table 2:** Detailed information of significantly changed lipids between endometriosis patients and healthy controls.

**Lipid molecular**	**Molecular formula**	**Adduct**	***m/z***	***t_*R*_* (min)**	***P*-values**	**VIP-values**	**Fold change**
**POSITIVE MODE**							
PC(18:1/22:6)	C_48_H_8_O_8_N_1_P_1_	M+H	832.5815	11.7155	0.012	1.1382	1.1120
PC(20:1/14:1)	C_42_H_80_O_8_N_1_P_1_	M+H	758.5690	12.7660	0.005	1.0215	1.6466
PC(20:3/20:4)	C_48_H_82_O_8_N_1_P_1_	M+H	832.5851	11.4469	0.03	2.8718	1.4716
**NEGATIVE MODE**							
PA(25:5/22:6)	C_50_H_77_O_8_P_1_	M–H	835.5275	11.9538	0.037	3.7783	1.6479
PS(20:3/23:1)	C_49_H_88_O_10_N_1_P_1_	M–H	880.6049	11.7765	0.028	2.1203	1.2424

## Discussion

Quantitative tracing of lipid metabolism under pathophysiological stimuli, environmental or genetic modifications has been successfully used as a diagnostic tool to monitor disease and develop new treatment strategies. In order to optimize lipidomics system, lipids extraction was performed according to MTBE method in this study. Compared to traditional methods, such as Bligh&Dyer method and Folch method, MTBE method delivers similar or better recoveries and performs better extraction efficiency, especially for PC, TG, and SM. Furthermore, MTBE is noncorrosive and chemically stable without forming peroxides during storage, which presents no danger to degrade labile lipids (Matyash et al., 2008). Previous studies reported alterations of lipid profile in serum, peritoneal fluid, follicular fluid, and endometrium of endometriosis patients (Vouk et al., 2012, 2016; Cordeiro et al., 2015; Chagovets et al., 2017). However, it is has not been investigated the alteration of lipid metabolism in endometrium.

This is the first time to report alterative levels of phosphatidic acid (PA), phosphatidylcholine (PC), and phosphatidylserine (PS) in eutopic endometrium of endometriosis patients at early stage. Instead of peripheral blood or urine, endometrium might contain various information to indicate the presence of endometriosis, using the direct source of the disease is perhaps logical to identify biomarkers for endometriosis (Ahn et al., 2017). Besides, pipelle biopsy is minimally invasive and suitable for outpatient. Hence, eutopic endometrium is ideal to analyze lipid profile of endometriosis for identifying potential biomarkers. We collected samples strictly on day 3–5 after menstrual cessation, which ensured all samples obtained in early follicle phase. The time to collect samples was chosen according to hysteroscopic surgical requirements and patient compliance. Unfortunately, we did not collect enough data on patients with advanced endometriosis because few patients in stage III–IV in our center met the inclusion criteria (exposure to hormonal drugs within 3 months).

In this study, we observed PA (25:5/22:6) was significantly higher in endometriosis patients compared to control group. It is the first to report PA is involved in the pathophysiology of endometriosis. PA is a phospholipid consisting out of a glycerol backbone with two fatty acids and one phosphate group attached, which is a central intermediate for the synthesis and storage of membrane lipids (Castro-Gomez et al., 2015). It has been implicated in various cellular signaling pathways, including in cell growth, proliferation, cell motility, and reactive oxygen species (ROS) production (Wang et al., 2006). PA has not only been shown to exert anti-apoptotic effects (Wang et al., 2006), but also has been identified as a mitogenic activator of the mammalian target of rapamycin signaling pathway to promote cell proliferation and generate survival signals (Chen, 2004), which might contribute to active proliferative capacity of endometriosis. Moreover, PA is related to cell motility (O'Luanaigh et al., 2002; Su et al., 2006), which may promote endometrium migration and invasion. PA is also responsible for the production of ROS via activating NADPH oxidase (Palicz et al., 2001). Excessive release of ROS plays an essential role in the inflammation process, which is involved in the pathogenesis of endometriosis by inducing endometrial fragment adhesion, proliferation, and neovascularization (Donnez et al., 2016). In addition, previous evidences suggested prostaglandin E2 (PGE2) and cyclooxygenase-2 (COX-2) in pathophysiology and pathogenesis of endometriosis (Wu et al., 2007; Banu et al., 2008; Machado et al., 2010). Lysophosphatidic acid (LPA) signaling stimulates PGE2 production and COX-2 expression in endometrial cells (Lin et al., 2008; Woclawek-Potocka et al., 2009). LPA is considered as potential factor for endometriosis (Ye and Chun, 2010), which is as the main source of PA. In this study, no significant difference of LPA was detected in endometrium. We speculated that PA alteration occurs earlier compared to LPA in the development of endometriosis, which might be used as a novel predictor of endometriosis at early stage.

Eutopic endometrium contributed to pathogenesis of endometriosis due to the increase of proliferation, migration, and invasion of ectopic endometrium (Joshi et al., 2015; Laudanski et al., 2015). We detected PS (20:3/23:1) decreased significantly in endometriosis group in this study. Only one study mentioned PS level was observed lower in follicular fluid in endometriosis patients previously (Cordeiro et al., 2015). Phospholipids are maintained asymmetrically in the eukaryotic plasma membrane in health cells. PS exposure on the cell surface shows an apoptotic signal for phagocytes (Segawa and Nagata, 2015; Nagata et al., 2016). Lower PS (20:3/23:1) levels may be due to reduced endometrial apoptotic cells in endometriosis patients. Three unsaturated PC (20:3/20:4), PC (18:1/22:6), PC (20:1/14:1) were significantly decreased in eutopic endometrium of endometriosis patients. Our results are consistent with previous studies (Vouk et al., 2012, 2016; Cordeiro et al., 2015). PC is one of the major sources of polyunsaturated fatty acids, that are the precursors of eicosanoids and has numerous biological activities (van der Veen et al., 2017). Evidence has accrued that PC contribute to both proliferative growth and programmed cell death (Ridgway, 2013). PC synthesis is increased in response to fatty acid and fatty acid-derived substrates, which is frequently observed in cancer cells (Ridgway, 2013). Unlike malignancy, PC markedly decreased in endometrium in endometriosis. This difference can be used to distinguish between endometriosis and malignancy. PC serves as a source for sphingomyelins and production of prostaglandins that may lead to decreased PC levels in endometrial tissue in this study. Sphingomyelins are abundant in endometriosis, which promotes cell survival in response to apoptotic stimuli (Vouk et al., 2012). Prostaglandins mediate inflammation in pathophysiology of endometriosis (Banu et al., 2008). Meanwhile, PC itself is also closely related to inflammation process (Drobnik et al., 2003; Ganna et al., 2014). Thus, PC has been correlated as a potential biomarker for endometriosis.

## Conclusion

This is the first report that PA, PC, and PS alterations in eutopic endometrium of endometriosis at stage I–II by UHPLC-ESI-HRMS-based metabolomics. These findings provide potential biomarkers for semi-invasive diagnose of endometriosis at early stages in clinical practice. However, a large sample size study on endometriosis lipidolomics analysis is needed to validate the implications of these individual lipids in the pathophysiology of endometriosis. Our findings also provide potential targets for therapeutic approach of this disease.

## Author contributions

JL: conceived of study, wrote the manuscript and supervised patient recruitment. YG, LG, HZ and DL: contributed to the study execution, analysis, and interpretation of data. XG: performed data analysis and interpretation. JS and PC: reviewed manuscript. HB, MH, ZM and XL: supervised patient recruitment, data collection, data evaluation, drafting, editing, and approving the final version of this paper for submission.

### Conflict of interest statement

The authors declare that the research was conducted in the absence of any commercial or financial relationships that could be construed as a potential conflict of interest.
